# Education level as a predictor of survival in patients with multiple myeloma

**DOI:** 10.1186/s12885-020-07178-5

**Published:** 2020-08-08

**Authors:** Limei Xu, Xiuju Wang, Xueyi Pan, Xiaotao Wang, Qing Wang, Bingyi Wu, Jiahui Cai, Ying Zhao, Lijuan Chen, Wuping Li, Juan Li

**Affiliations:** 1 Department of Hematology, The First Affiliated Hospital of Sun Yat-sen University, Guangzhou, Guangdong China; 2 Department of Hematology, Sun Yat-Sen Memorial Hospital of Sun Yat-Sen University, Guangzhou, Guangdong China; 3 Department of Hematology, The First Affiliated Hospital of Guangdong Pharmaceutical University, Guangzhou, Guangdong China; 4 Department of Hematology, The Second Affiliated Hospital of Guilin Medical University, Guilin, Guangxi China; 5 Department of Hematology, Guizhou Provincial People’s Hospital, Guiyang, Guizhou China; 6 Department of Hematology, Shunde Hospital of Southern Medical University, Shunde, Guangdong China; 7 Department of Hematology, First People’s Hospital of Foshan, Foshan, Guangdong China; 8 Department of Hematology, The First Affiliated Hospital With Nanjing Medical University, Nanjing, Jiangsu China; 9 Department of Internal Medicine, Jiangxi Tumor Hospital, Nanchang, Jiangxi China

**Keywords:** Education level, Sociodemographic status, Multiple myeloma, Survival prognosis

## Abstract

**Background:**

Disparities in multiple myeloma (MM) prognosis based on sociodemographic factors may exist. We investigated whether education level at diagnosis influenced Chinese MM patient outcomes.

**Methods:**

We performed a multicenter retrospective analysis of data from 773 MM patients across 9 centers in China from 2006 to 2019. Sociodemographic and clinical factors at diagnosis and treatment regimens were recorded, and univariate and multivariate analyses were performed.

**Results:**

Overall, 69.2% of patients had low education levels. Patients with low education levels differed from those with high education levels in that they were more likely to be older, and a higher proportion lived in rural areas, were unemployed, had lower annual incomes and lacked insurance. Additionally, compared to patients with high education levels, patients with low education levels had a higher proportion of international staging system (ISS) stage III classification and elevated lactate dehydrogenase (LDH) levels and underwent transplantation less often. Patients with high education levels had a median progression-free survival (PFS) of 67.50 (95% confidence interval (CI): 51.66–83.39) months, which was better than that of patients with low education levels (30.60 months, 95% CI: 27.38–33.82, *p* < 0.001). Similarly, patients with high education levels had a median overall survival (OS) of 122.27 (95% CI: 117.05–127.49) months, which was also better than that of patients with low education levels (58.83 months, 95% CI: 48.87–62.79, *p* < 0.001). In the multivariable analysis, patients with high education levels had lower relapse rates and higher survival rates than did those with low education level in terms of PFS and OS (hazard ratio (HR) = 0.50 [95% CI: 0.34–0.72], *p* < 0.001; HR = 0.32 [0.19–0.56], *p* < 0.001, respectively).

**Conclusions:**

Low education levels may independently predict poor survival in MM patients in China.

## Background

Multiple myeloma (MM) is characterized by the clonal proliferation of malignant plasma cells, causing lytic skeletal lesions, renal failure, hypercalcemia, and anemia, and patients typically present with monoclonal protein in the serum and/or urine [1, 2]. Currently, MM is the second-most common malignancy of the blood in many countries and has been estimated to account for 1.82% of all malignancies and 18% of all hematological malignancies, according to data from the United States [3, 4].

In recent years, with the continuous advent of new drugs and new treatments, the prognosis of patients with MM has been greatly improved. However, not all MM patients benefit equally from these improvements [5]. To explore the causes of this difference, a few studies from the Cancer Registry and the SEER database have shown the impact of racial and socioeconomic status (SES) disparities on the prognosis of patients with multiple myeloma [6–10]. Some studies have reported a significant increase in the risk of MM in individuals with low SES [8–10]. In addition, some studies reported differences in the clinical characteristics, incidence and survival prognosis among patients with MM across racial and ethnic groups [6], while some studies showed no consistent association between race/ethnicity or SES and survival outcomes after adjustment for confounders [7, 11, 12].

Globally, compared with the United States and other high-income countries, low- or middle-income countries have slower regulatory approval of drugs, fewer types of drugs available, and higher drug prices when adjusted for gross domestic product per capita; thus, the chances of effective treatment for these MM patients are greatly reduced [13, 14]. However, the mortality of MM in China, a country with a large population, has increased in recent years, especially in rural areas [15]. The impact of demographic and socioeconomic factors on the prognosis and survival of patients with MM has not been reported in developing countries such as China.

Education level is an important factor in patients’ demography. To understand the relationship between the education level and survival prognosis of Chinese MM patients, demographic factors (e.g., education level, occupational status, income, place of residence, marital status) and clinical characteristics (e.g., initial disease staging, lactate dehydrogenase (LDH) level, cytogenetics, comorbidities) at diagnosis and treatment regimens (e.g., underwent transplantation) were recorded and analyzed.

## Methods

### Patients

This retrospective, multicenter study was conducted in 9 centers across several provinces in China. A total of 773 newly diagnosed MM patients were enrolled in this study from January 2006 to July 2019 at each of the participating institutions. In accordance with the diagnostic criteria for multiple myeloma and disease progression, eligible patients were defined according to standard International Myeloma Working Group criteria [16, 17]. The treatment of patients was divided into transplantation and nontransplantation. Progression-free survival (PFS) was calculated from the time of the initial diagnosis of MM to disease progression, death or the last follow-up, and overall survival (OS) was calculated from the time of the initial diagnosis of MM until death or the last follow-u.

### Sociodemographic and clinical variables

We analyzed the personal information and clinical information of each patient at the time of the first visit, including age, sex, smoking status (yes or no), marital status (married, single, divorced or widowed), place of residence (urban or rural), the distance between place of residence and the hospital (in the same or different provinces), insurance status (insured or uninsured), and annual household income (<$42,500 USD, ≥$42,500 USD). As it costs approximately $42,500 USD to receive regular induced chemotherapy for 4 cycles and subsequent autologous stem cell transplantation (ASCT) for MM patients, we set $42,500 USD as the cut-off for annual household income. The education level was divided into two classes based on records of formal schooling: secondary school or lower was defined as a low education level, and a bachelor’s degree or higher was defined as a high education level. Occupational status was divided into employed and unemployed.

Clinical data included initial symptoms, comorbidity at the time of MM diagnosis, time from the onset of symptoms to diagnosis (< 1 month, ≥1 month), international staging system (ISS) stage (I, II, III), LDH level, and cytogenetic abnormality by fluorescence in situ hybridization (FISH). Briefly, translocation 4;14 [t (4;14)] and/or del [17p] and/or t [14;16] determined by FISH was defined as high risk cytogenetics; not carrying these mutations was defined as a standard risk cytogenetics [18]. The treatment includes whether to transplant or not. Treatment compliance was expressed by whether patients underwent regular treatment or not. The initial symptoms included bone pain, anemia, infection, anesthesia, and renal insufficiency.

### Statistical analysis

SPSS Statistics version 23 was utilized for statistical analysis. Patient baseline characteristics were analyzed using Student’s t-test or the chi-square test. The Kaplan-Meier method was performed for survival analysis, and differences were analyzed using the log-rank test. Univariate and multivariate analyses of features predicting survival were examined using hazard ratios (HRs) and corresponding 95% confidence intervals (95% CIs) calculated from Cox proportional hazards models. *p* < 0.05 was considered to be statistically significant.

## Results

### Baseline demographic and clinical characteristics of the MM patients

The main demographic, socioeconomic, and clinical features of the patients are listed in Table 1. Our cohort included 773 patients: 56.9% were male, 53.3% were under the age of 60, and 28.2% had a history of smoking. Most patients were married (96.0%), and most lived in the same province as their treating hospital and in urban areas (86.4 and 71.4%). Additionally, 69.2% of patients had low education levels, and only 18.6% were still employed during treatment. A total of 77.0% of patients had lower incomes (≤ $42,500 USD), and no insurance was listed for 69.3% of patients. The initial symptoms of the patients were mainly bone pain, followed by anemia and renal function impairment. Additionally, 38.5% of patients had cardiovascular disease and/or metabolic syndrome, and 4.7% had other tumors. The time from onset to definite diagnosis varied with most of the patients receiving a definite diagnosis after more than 1 month (76.6%), and 29.2% of the patients had ISS stage III disease at the time of onset. A total of 18.6% of the patients had LDH levels greater than 240 U/L, and 17.6% of the patients had high-risk cytogenetics. Moreover, 36.6% of patients underwent transplantation and 67.8% of the patients received regular treatment and underwent regular follow-up.

**Table 1:** Characteristics of the patients with multiple myeloma

	*N* = 773
Variables	N (%)
**Sex**	
Male	440 (56.9)
Female	333 (43.1)
**Age**	
< 60	412 (53.3)
≥ 60	361 (46.7)
**Smoking**	
Yes	218 (28.2)
No	555 (71.8)
**Marital status**	
Married	742 (96.0)
Unmarried	9 (1.2)
Divorced	13 (1.7)
Widowed	9 (1.1)
**Residential area**	
Urban	552 (71.4)
Rural	221 (28.6)
**Distance to hospital**	
In the same province	668 (86.4)
In a different province	105 (13.6)
**Education level**	
Low education level	535 (69.2)
High education level	226 (29.2)
Unknown	12 (1.6)
**Occupational status**	
Employed	144 (18.6)
Unemployed	605 (78.3)
Unknown	24 (3.1)
**Average annual income**	
≤ $42,500 USD	595 (77.0)
> $42,500 USD	117 (15.1)
Unknown	61 (7.9)
**Insurance status**	
Any insurance	174 (22.5)
No insurance	536 (69.3)
Unknown	63 (8.2)
**Initial symptoms**	
Bone pain	508 (65.7)
Anemia	118 (15.3)
Infection	34 (4.4)
Anesthesia	11 (1.4)
Renal insufficiency	47 (6.1)
Others	55 (7.1)
**Comorbidity**	
None	274 (35.5)
Cardiovascular disease and/or metabolic syndrome	298 (38.5)
Other tumors	36 (4.7)
Other	198 (25.6)
**Time to diagnosis**	
≤1 month	181 (23.4)
> 1 month	592 (76.6)
**ISS stage**	
I/II	531 (68.7)
III	226 (29.2)
Unknown	16 (2.1)
**LDH level**	
< 240 U/L	565 (73.1)
≥240 U/L	144 (18.6)
Unknown	64 (8.3)
**Cytogenetic abnormality by FISH**	
High risk	136 (17.6)
Standard risk	429 (55.5)
Unknown	208 (26.9)
**Receipt of transplant**	
Yes	283 (36.6)
No	490 (63.4)
**Regular treatment**	
Yes	524 (67.8)
No	249 (32.2)

*ISS* international staging system, *LDH* lactate dehydrogenase, *FISH* fluorescence in situ hybridization

### Comparison between MM patients with a high vs low education level

Information on education level was available for 98.5% of the patients (761/773). Patients with low education levels were more likely to be older (≥60 years, 51.2% vs 36.3%, *p* < 0.001), and a higher proportion were female (46.9% vs 35.0%, *p* = 0.002), lived in rural areas (39.1% vs 5.3%, *p* < 0.001), were unemployed (86.9% vs 66.2%, *p* < 0.001), had a lower income (94.5% vs 59.3%, *p* < 0.001), lacked insurance (82.2% vs 60.4%, *p* < 0.001) and had comorbidities (32.3% vs 43.8%, *p* = 0.003). Additionally, time to diagnosis > 1 month was more frequent in patients with low education levels (81.3% vs 65.0%, *p* < 0.001), and they consistently had a higher ISS stage (III, 32.5% vs 23.7%, *p* = 0.014) and elevations in LDH levels (≥240 U/L, 23.1% vs 13.0%, *p* = 0.003). However, there was no difference in cytogenetics between the two groups. In addition, patients with high education levels were more likely to be treated via transplantation (59.3% vs 27.9%, *p* < 0.001) and undergo regular treatment (87.6% vs 60.7%, *p* < 0.001) than patients with low education levels (Table 2).

**Table 2:** Comparison of demographic and clinical characteristics between patients with high and low education levels

Variables	Low education level		High education level		*P*
	*N* = 535	(%)	*N* = 226	(%)	
**Age**					< 0.001
< 60	261	48.8	144	63.7	
≥ 60	274	51.2	82	36.3	
**Sex**					0.002
Male	284	53.1	147	65.0	
Female	251	46.9	79	35.0	
**Smoking**					0.183
Yes	158	29.5	56	24.8	
No	377	70.5	170	75.2	
**Marital status**					0.657
Married	515	96.3	216	95.6	
Other	20	3.7	10	4.4	
**Residential area**					< 0.001
Urban	326	60.9	214	94.7	
Rural	209	39.1	12	5.3	
**Distance to hospital**					0.287
Same province	458	85.6	200	88.5	
Different province	77	14.4	26	11.5	
**Occupational status**					< 0.001
Employed	68	13.1	76	33.8	
Unemployed	453	86.9	149	66.2	
**Average annual income**					< 0.001
< $42,500 USD	464	94.5	131	59.3	
≥ $42,500 USD	27	5.5	90	40.7	
**Insurance status**					< 0.001
Any insurance	88	17.8	86	39.6	
No insurance	405	82.2	131	60.4	
**Comorbidity**					< 0.003
Yes	362	67.7	127	56.2	
No	173	32.3	99	43.8	
**Time to diagnosis**					< 0.001
≤ 1 month	100	18.7	79	35.0	
> 1 months	435	81.3	147	65.0	
**ISS stage**					0.014
I/II	351	67.5	172	76.4	
III	169	32.5	53	23.6	
**LDH**					
≥ 240 U/L	115	23.1	26	13.0	0.003
< 240 U/L	383	76.9	174	87.0	
**Cytogenetics**					0.768
High risk	96	24.5	38	23.3	
Standard risk	296	75.5	125	76.7	
**Receipt of transplant**					< 0.001
Yes	149	27.9	134	59.3	
No	386	72.2	92	40.7	
**Regular treatment**					< 0.001
Yes	325	60.7	198	87.6	
No	210	39.3	28	12.4	

*ISS* international staging system, *LDH* lactate dehydrogenase

### Univariate analyses for PFS and OS

**Fig. 1:**
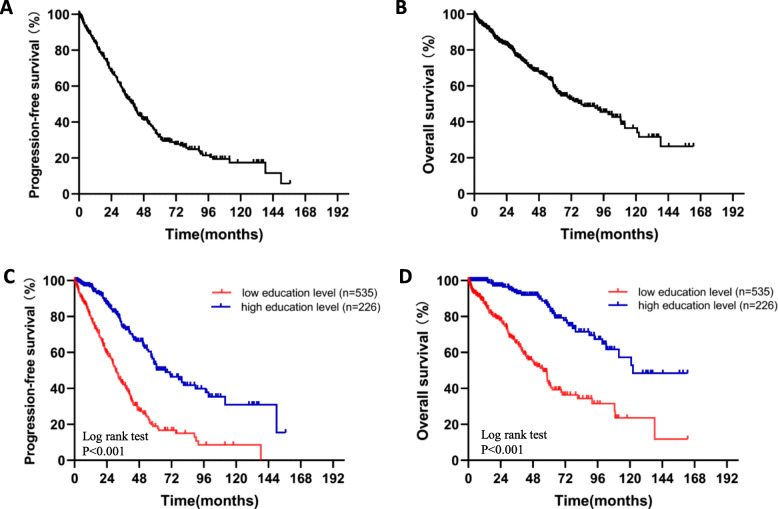
Kaplan-Meier plots of PFS and OS for MM patients. **a** The median PFS for 773 MM patients. **b** The median OS for 773 MM patients. **c** Kaplan-Meier plots of PFS were compared between MM patients with high and low education levels. **d** Kaplan-Meier plots of OS were compared between MM patients with high and low education levels

The median follow-up for the entire cohort was 29.6 months (range, 0.3 months to 162.8 months) from the start of diagnosis. Kaplan-Meier analyses showed that the median PFS and OS for all patients were, respectively, 39.93(95% CI: 35.79–44.07) months and 79.63 (95% CI: 58.88–100.48) months (Fig. 1a, b). Patients with high education levels had a median PFS of 67.50 (95% CI: 51.66–83.39) months, which was better than that of patients with low education levels (30.60 months, 95% CI: 27.38–33.82, *p* < 0.001, Fig. 1**c**). Similarly, patients with high education levels had a median OS of 122.27 (95% CI: 117.05–127.49) months, which was also better than that of patients with low education levels (58.83 months, 95% CI: 48.87–62.79, *p* < 0.001, Fig. 1**d**).

In this study, univariate Cox regression analyses were performed to explore the association between the baseline factors of patients and PFS and OS. The sociodemographic factors associated with worse PFS and OS in the univariate Cox regression model included age (HR = 1.04 [95% CI: 1.02–1.04]; HR = 1.03[95% CI: 1.02–1.05], respectively), residence in a rural setting (HR = 1.48[95% CI: 1.14–1.93]; HR = 1.47[95% CI: 1.06–2.05], respectively), living in a different province from the treating hospital (HR = 1.18[95% CI: 1.01–1.37]; HR = 1.15[95% CI: 0.94–1.41], respectively), being unemployed (HR = 1.67[1.22–2.30]; HR = 2.53[1.55–4.13], respectively), and a lack of insurance (HR = 1.54[95% CI: 1.15–2.06]; HR = 2.16[95% CI: 1.43–3.29], respectively). Additional clinical factors associated with worse PFS and OS included complications at diagnosis (HR = 1.72[95% CI: 1.35–2.18]; HR = 2.54[95% CI: 1.81–3.56], respectively), time to diagnosis > 1 month (HR = 1.47[95% CI: 1.13–1.91]; HR = 1.96[95% CI: 1.37–2.81], respectively), ISS stage III disease (HR = 1.23[95% CI: 1.09–1.39]; HR = 1.38[95% CI: 1.19–1.60], respectively), elevations in LDH levels (HR = 1.87[95% CI: 1.43–2.46]; HR = 1.85[95% CI: 1.32–2.60], respectively), high-risk cytogenetics (HR = 1.68[95% CI: 1.26–2.25]; HR = 1.98[95% CI: 1.38–2.82], respectively), no transplantation (HR = 2.98[95% CI: 2.34–3.80]; HR = 2.53[95% CI: 1.87–3.44], respectively), and irregular treatment (HR = 3.28[95% CI: 2.59–4.16]; HR = 3.51[95% CI: 2.61–4.71], respectively). In addition, sociodemographic factors associated with better PFS and OS in the univariate Cox regression model included a high education level (HR = 0.39[95% CI: 0.30–0.52]; HR = 0.25[95% CI: 0.17–0.38], respectively) and a high annual income (i.e., ≥ $42,500; HR = 0.51[95% CI: 0.37–0.70]; HR = 0.36[95% CI: 0.23–0.55], respectively) (Table 3).

**Table 3:** Univariate analysis of the baseline parameters associated with PFS and OS

Variable	PFS		OS	
	HR (95% CI)	*P*	HR (95% CI)	*P*
**Sex**				
Male vs female	1.04 (0.83–1.30)	0.74	0.97 (0.73–1.29)	0.823
**Age** (per year of age)	1.03 (1.02–1.04)	< 0.001	1.04 (1.02–1.05)	< 0.001
**Smoking**				
Yes vs no	0.95 (0.74–1.23)	0.71	0.93 (0.68–1.29)	0.671
**Marital status**				
Married vs other	0.78 (0.44–1.34)	0.41	0.85 (0.40–1.81)	0.672
**Residential area**				
Rural vs urban	1.48 (1.14–1.93)	0.003	1.47 (1.06–2.05)	0.023
**Distance to hospital**				
Different province vs the same province	1.18 (1.01–1.37)	0.039	1.15 (0.94–1.41)	0.175
**Education level**				
High vs low education level	0.39 (0.30–0.52)	< 0.001	0.25 (0.17–0.38)	< 0.001
**Occupational status**				
Unemployed vs employed	1.67 (1.22–2.30)	0.002	2.53 (1.55–4.13)	< 0.001
**Average annual income**				
≥ $42,500 vs < $42,500 USD	0.51 (0.37–0.70)	< 0.001	0.36 (0.23–0.55)	< 0.001
**Insurance status**				
No insurance vs any insurance	1.54 (1.15–2.06)	0.004	2.16 (1.43–3.29)	< 0.001
**Comorbidity**				
Yes vs no	1.72 (1.35–2.18)	< 0.001	2.54 (1.81–3.56)	< 0.001
**Time to diagnosis**				
> 1 vs ≤1 month	1.47 (1.13–1.91)	0.004	1.96 (1.37–2.81)	< 0.001
**ISS stage**				
III vs I/II	1.23 (1.09–1.39)	0.001	1.38 (1.19–1.60)	< 0.001
**LDH level**				
≥ 240 vs < 240 U/L	1.87 (1.43–2.46)	< 0.001	1.85 (1.32–2.60)	< 0.001
**Cytogenetics**				
High risk vs standard risk	1.68 (1.26–2.25)	< 0.001	1.98 (1.38–2.82)	< 0.001
**Receipt of transplant**				
No vs yes	2.98 (2.34–3.80)	< 0.001	2.53 (1.87–3.44)	< 0.001
**Regular treatment**				
no vs yes	3.28 (2.59–4.16)	< 0.001	3.51 (2.61–4.71)	< 0.001

*PFS* progression-free survival, *OS* overall survival, *HR* hazard ratio, *CI* confidence interval, *ISS* international staging system, *LDH* lactate dehydrogenase

### Multivariate analyses for PFS and OS

To further analyze the influence of sociodemographic factors on patient survival, multivariate Cox regression analyses were conducted. Since age is an important factor affecting survival and we found that education and age have interactive effects on survival, we analyzed the effects of demographic and clinical factors on PFS and OS in patients with MM by dividing them into groups of patients < 60 years old and **≥** 60 years old.

We found that in different age groups, education level, LDH levels, cytogenetics and receipt of transplant were independently associated with PFS, while in the age stratification analysis, regular treatment was an independent factor affecting the PFS of patients < 60 years old. (Table 4). In addition, for all patients, the independent risk factors affecting OS included patients` age (per year of age), low education level, elevated LDH level, high-risk cytogenetics, complications at diagnosis and irregular treatment. In the analysis of age stratification, for patients younger than 60 years old, education level, cytogenetics and regular treatment were independent prognostic factors for OS. Additionally, for patients ≥60 years old, education level, LDH levels, cytogenetics and complications at diagnosis were independent prognostic factors for OS (Table 5).

**Table 4:** Multivariate analysis of baseline parameters associated with PFS

Variables	PFS	
	HR (95% CI)	*P*
**All patients**		
Education level: high vs low	0.50 (0.34–0.72)	< 0.001
LDH: ≥240 vs < 240 U/L	2.08 (1.48–2.94)	< 0.001
Cytogenetics: high risk vs standard risk	1.77 (1.28–2.45)	0.001
Receipt of transplant: no vs yes	2.70 (1.95–3.74)	< 0.001
**Patients < 60 years**		
Education level: high vs low	0.47 (0.29–0.74)	0.002
LDH: ≥240 vs < 240 U/L	2.45 (1.52–3.95)	0.001
Cytogenetics: high risk vs standard risk	1.85 (1.18–2.90)	0.007
Receipt of transplant: no vs yes	2.00 (1.20–3.35)	0.008
Regular treatment: no vs yes	2.08 (1.53–3.73)	0.015
**Patients** ≥ **60 years**		
Education level: high vs low	0.53 (0.29–0.98)	0.043
LDH: ≥240 vs < 240 U/L	1.81 (1.10–3.00)	0.020
Cytogenetics: high risk vs standard risk	1.68 (1.03–2.72)	0.037
Receipt of transplant: no vs yes	2.38 (1.36–4.17)	0.002

*PFS* progression-free survival, *HR* hazard ratio, *CI* confidence interval, *LDH* lactate dehydrogenase

**Table 5:** Multivariate analysis of baseline parameters associated with OS

Variables	OS	
	HR (95% CI)	*P*
**All patients**		
Age (per year of age)	1.03 (1.00–1.05)	0.028
Education level: high vs low	0.32 (0.19–0.56)	< 0.001
LDH: ≥240 vs < 240 U/L	1.86 (1.18–2.94)	0.008
Cytogenetics: high risk vs standard risk	2.01 (1.32–3.06)	0.001
Comorbidity: yes vs no	2.01 (1.25–3.23)	0.004
Regular treatment: no vs yes	1.73 (1.08–2.77)	0.024
**Patients < 60 years**		
Education level: high vs low	0.30 (0.14–0.62)	0.001
Cytogenetics: high risk vs standard risk	2.37 (1.30–4.32)	0.005
Regular treatment: no vs yes	2.17 (1.08–4.38)	0.030
**Patients** ≥ **60 years**		
Education level: high vs low	0.26 (0.11–0.62)	0.002
LDH: ≥240 vs < 240 U/L	2.27 (1.24–4.18)	0.008
Cytogenetics: high risk vs standard risk	1.84 (1.01–3.33)	0.045
Comorbidity: yes vs no	3.16 (1.32–7.55)	0.010

*OS* overall survival, *HR* hazard ratio, *CI* confidence interval, *LDH* lactate dehydrogenase

## Discussion

To the best of our knowledge, this study is the first to examine the relationship between sociodemographic factors and survival in patients with MM in China. The prognostic factors of MM mainly include host factors, tumor characteristics and treatment methods [19]. A single factor is often not enough to determine the prognosis. Among the tumor factors, we usually evaluate the prognosis of patients by ISS stage, LDH level and cytogenetics. Moreover, in terms of treatment, we also found that hematopoietic stem cell transplantation in patients with MM can significantly improve the survival prognosis [20]. However, there is no consensus on the impact of patient host factors on prognosis. To date, the prognosis of patients has not been evaluated with these three factors at the same time. Therefore, we included demographic factors (e.g., age, sex, education level, income, work, insurance), tumor characteristics (e.g., ISS stage, cytogenetics, LDH level) and treatment methods in the analysis.

SES is often measured by income, education or occupation, either as singular variables or in combination, which is a strong predictor for survival prognoses in MM as well as other diseases [6, 8, 21, 22]. It can be assumed that the education level covaries with SES. Cancer death rates vary considerably by level of education [23]. Attalla, K. et al. found that penile cancer patients with low education levels were more likely to be diagnosed with a worse pathologic T stage [24]. Hwang, K.T. et al. found that high education levels conferred a superior prognosis for breast cancer patients in the subgroup aged > 50 years; these patients had a lower mean age at the first diagnosis and more favorable biological features [25].

In our study, we set income, education level and occupational status as independent factors. As age and educational level of these patients have an interactive effect on survival, we conducted a hierarchical analysis of age. The results of multivariate Cox regression analyses showed that education level was an independent factor affecting the prognosis of MM patients after adjustments were made for potential confounders. Our results showed that patients with high education levels were more likely to have a longer PFS and OS. Patients with high education levels were younger, and the time from onset of symptoms to diagnosis was shorter. Those factors may result in patients in this subgroup having lower tumor loads (e.g., LDH levels and ISS stages) and fewer complications. In addition, patients with high education levels were more likely to choose effective treatments, such as transplantation, than patients with low education levels, and these patients more often received regular treatment. Therefore, the above factors may partly explain why education levels affect patient survival.

In addition, our results showed that patients with high education levels have financial and work support, and they tend to have more stable employment and income. These factors may allow them to make treatment choices without cost restrictions and pay more attention to the efficacy of drugs so as to choose a more positive and effective treatment. Similarly, Alter, D.A. et al. reported that compared to patients with lower SES, more affluent or better educated patients were more likely to undergo active and effective treatment [26]. Additionally, insurance is also a very important economic factor, and we found that patients with high education levels are more likely to have insurance coverage. Several studies have reported that insurance status was associated with OS, and patients who were uninsured had poorer survival than those who were insured [7, 27, 28].

However, for patients with malignant tumors, the mechanism of the impact of education level on their survival is extremely complex. Linder, G. et al. found that high education levels were associated with a greater probability of being offered curative treatment and improved survival in esophageal and gastroesophageal junctional cancer in Sweden; the reason may be communication difficulties and a lack of understanding of treatment, which were more commonly reported in groups with low education levels [29]. This finding reflects that a high level of education can help patients gain a full understanding of their diseases and make it easier to acquire health-related knowledge. Additionally, our study showed that patient education levels were related to treatment compliance, and there was also one report showed that patients with a high education level have better treatment compliance [30]. Besides, some studies have shown that low education levels might undermine the patient’s initiative to seek healthcare services, leading to a delay in the diagnosis of a primary disease or a life-threatening complication [31, 32]. These factors also need to be fully taken into account.

Moreover, patient treatment can be managed according to their SES. At present, new drugs (such as bortezomib and lenalidomide) and ASCT can significantly improve survival in patients with MM, but these methods result in a great increase in the cost of treatment [33]. Therefore, drug-induced sequential ASCT is preferred for patients with high SES who are suitable for transplantation, and new drugs are preferred for patients with high SES who are not suitable for transplantation, while patients with low SES can choose less expensive options, such as regimens containing thalidomide combined with cyclophosphamide and dexamethasone. Palliative treatment is more suitable for patients with severe complications who cannot tolerate chemotherapy than for patients with low SES.

Our research has some limitations owing to its retrospective nature. In addition, some of the values were missing, but the proportion of missing values for most variables was less than 10%. In addition, we did not get the specific treatment details of these patients and there were many confounding variables in this study. In the future, we can further analyze the relationship between the specific treatment regimens, treatment response, comorbidities and educational levels and survival prognosis.

## Conclusions

With continuous advancements in the treatment of multiple myeloma, the prognosis of patients has greatly improved. However, not all patients benefit equally. By analyzing the relationship between sociodemographic factors and the survival of patients with multiple myeloma in China, we found that education level is an independent factor affecting survival outcomes. In particular, MM patients with high education levels have a better economic foundation, can seek medical treatment in a more timely manner, can choose the best treatment regimens and can be treated more regularly. Therefore, the results of this study indicate that we can use the education level of newly diagnosed patients to evaluate the prognosis of these patients and to create more reasonable treatment plans.

## Data Availability

The datasets generated and/or analysed during the current study are not publicly available as presently we have not been granted permission by the institutional review board to do so. However, data can be made available from the corresponding author on reasonable request.

## Abbreviations

MM: Multiple myeloma
ISS: International staging system
LDH: Lactate dehydrogenase
PFS: Progression-free survival
OS: Overall survival
SES: Socioeconomic status
HR: Hazard ratio
95% CI: 95%confidence interval
FISH: Fluorescence in situ hybridization
